# Subjective Impressions Do Not Mirror Online Reading Effort: Concurrent EEG-Eyetracking Evidence from the Reading of Books and Digital Media

**DOI:** 10.1371/journal.pone.0056178

**Published:** 2013-02-06

**Authors:** Franziska Kretzschmar, Dominique Pleimling, Jana Hosemann, Stephan Füssel, Ina Bornkessel-Schlesewsky, Matthias Schlesewsky

**Affiliations:** 1 Department of English and Linguistics, Johannes Gutenberg-University, Mainz, Germany; 2 Institute of Book and Media Studies Johannes Gutenberg-University, Mainz, Germany; 3 Department of German Philology, Georg August-University, Göttingen, Germany; 4 Department of Germanic Linguistics, University of Marburg, Marburg, Germany; Centre national de la recherche scientifique, France

## Abstract

In the rapidly changing circumstances of our increasingly digital world, reading is also becoming an increasingly digital experience: electronic books (e-books) are now outselling print books in the United States and the United Kingdom. Nevertheless, many readers still view e-books as less readable than print books. The present study thus used combined EEG and eyetracking measures in order to test whether reading from digital media requires higher cognitive effort than reading conventional books. Young and elderly adults read short texts on three different reading devices: a paper page, an e-reader and a tablet computer and answered comprehension questions about them while their eye movements and EEG were recorded. The results of a debriefing questionnaire replicated previous findings in that participants overwhelmingly chose the paper page over the two electronic devices as their preferred reading medium. Online measures, by contrast, showed shorter mean fixation durations and lower EEG theta band voltage density – known to covary with memory encoding and retrieval – for the older adults when reading from a tablet computer in comparison to the other two devices. Young adults showed comparable fixation durations and theta activity for all three devices. Comprehension accuracy did not differ across the three media for either group. We argue that these results can be explained in terms of the better text discriminability (higher contrast) produced by the backlit display of the tablet computer. Contrast sensitivity decreases with age and degraded contrast conditions lead to longer reading times, thus supporting the conclusion that older readers may benefit particularly from the enhanced contrast of the tablet. Our findings thus indicate that people's subjective evaluation of digital reading media must be dissociated from the cognitive and neural effort expended in online information processing while reading from such devices.

## Introduction

Reading is becoming an increasingly digital pastime. In the United States and the United Kingdom, sales of electronic books (e-books) have already overtaken those of print books and are continuing to increase rapidly (see Text S1 in the Supporting Information for additional details). Though readers in other countries (e.g. Germany) appear to be somewhat more conservative in their choice of reading media, the trend towards e-books nevertheless appears universal. Scientific texts, too, are progressing towards exclusively digital dissemination, with many journals – such as PLOS ONE – appearing only in electronic form, and many of the leading international scientific organisations (e.g. Max Planck Society, Wellcome Trust) endorsing the use of digital media in combination with open access to further the dissemination of scientific publications (see, for example, the 2003 Berlin Declaration on Open Access to Knowledge in the Sciences and Humanities (http://oa.mpg.de/lang/en-uk/berlin-prozess/berliner-erklarung/).

Nevertheless, many people continue to harbour a certain degree of skepticism towards reading on digital media. Thus, though digital book sales doubled from 2010 to 2011 in Germany, readers were still reluctant to accept e-books and e-book sales accounted for only 1% of overall book sales in Germany in 2011 (http://www.boersenverein.de/ebookstudie; see also Text S1 in the Supporting Information). This general sense of distrust towards new technologies is also a popular topic in the media: Germany's national tabloid Bild, for example, posed the question ‘Macht uns die moderne Technik dumm?’ ('Is modern technology making us stupid?'; Bild, 13.08.2012) and one of Germany's largest national newspapers, the Süddeutsche Zeitung, dedicated an entire weekend supplement to the potential impact of digital technology on child development (11./12.08.2012).

But from whence does this general reluctance to accept digital reading media stem? A large-scale survey of students and faculty at University College London [1] provides a tentative answer to this question. While the users of e-books (n = 760; 53% of the overall number of participants in the survey) praised their convenience, up-to-dateness and availability, they nevertheless judged ease of reading to be considerably worse than for conventional printed books.

It, however, remains an open question whether these subjective judgements of reading effort can be corroborated by objective measures. Indeed, it is a well-known observation within cognitive (neuro-)science that people's perception of their own behaviour does not always mirror neural activity or even fast and automatic measures of behaviour (e.g. eye movements during reading). As an example of the latter, consider the well-known phenomenon that sentences with transposed letters appear easy to read:

‘[A] widely circulated statement on the Internet claimed that resarceh at Cmabrigde Uinervtisy fuond that sentecnes in whcih lettres weer transpsoed (or jubmled up), as in the setnence you are now raeding, were easy to read and that letter position in words was not important to the ability to read successfully.’

Rayner, White, Johnson and Liversedge [2]

In contrast to this claim (which was actually a hoax and did not reflect the results of any research carried out at the University of Cambridge [2]) – and to our own subjective perception of relative ease when reading texts with transposed letters – a number of studies using eyetracking to provide an objective quantitative record of eye movements during reading have shown that there is, in fact, a cost to reading such texts (for a review, see[3]). Thus, even though participants report that they can read texts with transposed letters and comprehension tasks show that they can understand them, eye movement measures nevertheless reveal longer fixation times in comparison to normal texts. Similarly, findings from the electrophysiological domain show that sentences that are easy to comprehend and are judged to be highly acceptable can nevertheless engender increased local processing effort as indexed by event-related brain potentials (ERPs) [4]–[6]. In these cases, participants are not aware of any difficulty during online sentence comprehension (see also [7] for evidence of non-detected word stimuli modulating ERP correlates of language processing).

In light of these previous observations regarding the dissociability of conscious judgements and online processing as measured by means of electrophysiology and eyetracking, the present study aimed to examine whether people's comparatively negative subjective impressions regarding the readability of digital texts can be corroborated by objective quantitative measures of online processing effort using the two methods that currently provide the best established real time measures in reading research and language processing in general (EEG and eyetracking, see for example [8], [9]). By means of concurrent EEG and eyetracking measures, we examined EEG frequency activity and fixation durations while adults in two age groups (young and elderly) read texts on three different media (book page, tablet computer, e-reader) and answered comprehension questions about them. While the results of a debriefing questionnaire replicated previous findings in that participants showed a strong preference for reading from paper as opposed to a digital medium, online measures showed a different result. For young adults, neither fixation durations nor EEG theta band activity differed between the three media types. For older adults, by contrast, reading on a tablet computer elicited shorter fixation durations and lower theta band voltage density measures, thus suggesting an advantage for online reading, perhaps due to a higher degree of visual discriminability during information processing.

## Materials and Methods

As already noted above, the present study employed eyetracking and EEG to obtain quantifiable measures of online reading effort. Eyetracking has been used to inform reading research for over 30 years and the eye movement record (including, in particular, fixation durations) is assumed to provide a rather direct window onto the underlying cognitive processes involved [10]. Eye movement research has further been highly influential in the development of cognitive models of language comprehension (e.g. [11]–[16]) and reading (e.g. [17]–[19]). EEG-based research of language processing has been similarly influential, beginning with Kutas and Hillyard's [20] discovery of the N400 as an electrophysiological response to semantically unexpected words in a reading task. In addition to informing neurocognitive models of language processing by way of language-related event-related brain potentials (ERPs) (e.g. [21]–[25]), EEG has also been used to obtain measures within the frequency domain to provide a complementary perspective on cognitive processing related to language (e.g. [26]–[30]).

In recent years, the combination of eyetracking and EEG within a concurrent measurement setup has been shown to be methodologically feasible and a potential means of gaining new insights on the neurocognitive bases of natural reading [31]–[35]. (Note that the acquisition of concurrent EEG and eye movement measures was long considered impossible because eye movements lead to artefacts in the EEG signal.) In the present study, we capitalised upon this methodological advance in order to obtain both neurophysiological measures (EEG) and rapid, highly automatic behavioural responses (eye movements) as a function of information processing in reading from digital media as opposed to a traditional paper page.

On account of the experimental design employed (see below), which aimed to examine global (text-based) rather than local (event-related) measures of reading effort, we used EEG activity in the frequency rather than the time domain as our dependent electrophysiological measure (for a more general motivation for combining eye movement measures with EEG frequency analyses rather than ERPs, see [24]). Specifically, we analysed modulations of the theta frequency band, which has been linked to the encoding and retrieval of new information in working memory (for a review, see [36]). Modulations of theta activity have also been observed during reading tasks [26] and, in this context, particularly in response to syntactic processing demands [27]. Hence, theta activity has been demonstrated to reflect online demands of information processing in language comprehension.

### Participants

Thirty-six younger adults (18 females), mainly students at the University of Mainz (mean age: 25.7 years, range: 21–34), and twenty-one older adults, mostly retired senior citizens, (13 females, mean age: 66.8 years, range: 60–77) participated in the experiment. They were paid 7€ (younger adults) or 8€ per hour (older adults) for their participation. Participants were right-handed as assessed by a German version of the Edinburgh handedness inventory [37] and had normal or corrected-to-normal vision. None of them reported neurological or psychological disorders. All participants were naïve with respect to the purpose of the study. One of the participants in the younger group was excluded from the final data analysis due to problems in performing the task.

Note that a pilot study revealed that our experimental setup was incompatible with progressive bi- or trifocal glasses, because parts of the texts would not fall within the focal area used for reading. We therefore opted to not collect data from older participants who only had progressive multifocal glasses. Of the 21 older participants, two reported uncorrected vision, one participant used contact lenses, 8 participants had glasses and 10 participants used reading glasses. Among the younger participants, 24 participants reported uncorrected vision, 9 participants used glasses, and 2 participants used contact lenses.

The experiment was performed in accordance with the ethical standards laid down in the Declaration of Helsinki. Participants gave written informed consent before the beginning of the experiment and were informed that they could discontinue the study at any time should they wish to do so.

### Materials

The materials consisted of nine short texts of three different types (three scientific texts [38]–[40], 3 non-fiction texts [41]–[43], and 3 fiction texts [44]); all texts are available from the corresponding author upon request). The texts were adapted to be of similar length (mean length: 222 words, range: 176–266). Different text types were chosen in order to introduce a certain variability in the degree of reading effort required.

To allow for comparability across the three reading devices, the short texts were segmented into three pages for all devices. The first two pages of a text always contained 13 lines of text, while the third page differed from 2 through 13 lines. All texts were presented in Courier New font with identical font size, line spacing, and with left-justification. Line breaks were identical for all media so that the number of words per line was also held constant. Thus, this layout ensured that there would be the same amount of text on each page for each reading device and that the frequency of page turning was also identical across the devices. Small differences in font size resulting from different display resolutions were compensated by adjusting the distance between the eyes and the respective reading device. In addition, the distances (see below) were chosen to render available the same amount of letters per degree of visual angle.

Each participant read each text once and on only one reading device (i.e. three texts per device, with one text of each type). Thus, all participants read nine texts in total, with the assignment of individual texts to reading devices counterbalanced across participants.

### Data acquisition

The EEG was recorded from seventeen Ag/AgCl scalp electrodes (EasyCap GmbH, Herrsching, Germany), which were positioned according to the international 10–20 system (Fz, F3, F4, F7, F8, Cz, C3, C4, T7, T8, Pz, P3, P4, P7, P8, O1, O2, ground: AFz), and the impedances were kept below 5 kΩ. The electrooculogram (EOG) was recorded from three bipolar pairs of electrodes placed at the outer canthi of the eyes and above and below each eye. The EEG was referenced on-line to the right-mastoid electrode and amplified using a BrainAmp amplifier (Brain Products GmbH, Gilching, Germany) with a sampling rate of 1000 Hz.

Eye movements were recorded with an EyeLink 1000 tower-mounted eye-tracker (SR Research, Kanata, Canada). The sampling rate was 1000 Hz. Viewing was binocular, but only the right eye was recorded. For one elderly participant, we tracked the left eye as the signal on the right eye did not appear stable.

The stimuli were presented on one of three displays: a tablet computer (iPad 2; Apple Inc., Cupertino, CA), an e-reader (Kindle 3 (Keyboard); Amazon.com, Inc., Seattle, WA) and on a white paper page. Each device was positioned at an individual distance to the participant so that 2.7 letters always subtended 1° of visual angle. The approximate distances between display surface and participant were as follows: 44 cm (iPad), 40 cm (paper page), and 35 cm (Kindle). The room was brightly illuminated. Room lighting was the same for all three media and was chosen so that reading on the paper page and the Kindle was perceived as comfortable.

The resolutions on the three reading devices were as follows: iPad: 1024×768 (native resolution); Kindle: 800×600; paper: 4760×2700. Regarding luminance of the iPad 2, we used the maximum luminance provided by the device, namely 410 cd/m^2^ for peak brightness and 0.43 cd/m^2^ for black-level brightness (values taken from http://www.displaymate.com/iPad_2_ShootOut.htm). This amounts to a contrast ratio of 1.00 as calculated via the Michelson definition [C = (L_max_−L_min_)/(L_max_+L_min_); with C = contrast, L_max_ = maximal luminance, and L_min_  =  minimal luminance]. We used the Michelson definition of contrast because this has been applied in previous studies examining the impact of contrast on reading rates [45]. In order to determine the actual contrast ratios during the experiment, we measured luminance for black (minimal luminance) and white (maximal luminance) displays for each of the three media from the position of the participant (i.e. vertically level with the tower mount of the Eyelink 1000 and approximately 2 cm below the forehead rest) at the exact lighting settings used during data acquisition (device used for luminance measurements: Extech EA33 EasyView Light Meter; Extech Instruments, Nashua, NH). Michelson contrast ratios for the three devices were as follows: iPad: 0.48 (l µm_max_: 13.09 cd/m^2^, lum_min_: 4.58 cd/m^2^); Kindle: 0.01 (l µm_max_: 2.93 cd/m^2^, l µm_min_: 2.86 cd/m^2^); paper: 0.05 (l µm_max_: 4.09 cd/m^2^, l µm_min_: 3.72 cd/m^2^).

Regarding display size, we used an identical bounding box across devices (i.e. a frame within which the texts were displayed). The following values give the size of the bounding boxes for each reading device. The first value (UL) represents the upper left pixel position (x, y), the second value (BR) represents the lower right pixel position (x,y): iPad: UL (50,50), BR (800,622); Kindle: UL (25,15), BR (775, 587); paper: UL (500,100), BR (3700, 2520).

### Procedure

After arriving in the lab, participants were given general information about the experiment and gave written consent to participate. After electrode preparation, all participants were given a detailed written instruction about the procedure. The experiment was always run with two experimenters. Prior to and after the experiment, we measured a rest EEG. Participants sat comfortably on a chair (two minutes with their eyes closed and two minutes with open eyes, focusing on a fixation asterisk). The rest EEG was used to determine the individual alpha frequency (IAF) of each participant (see below).

Each experimental session started with a short practice period, in which participants read one non-fiction text once on a paper page and once on the Kindle in order to familiarise themselves with the procedure. The text was not presented in the actual experiment, but was similar in length to the experimental texts and identical to them in terms of layout.

The experiment began with a 13 point-grid calibration, which was repeated after a break or when deemed necessary by the experimenters. Participants read three texts on each display, one of each text type. The order of presentation on a display and the order of presentation of text types were pseudo-randomised using a Latin Square design. Thus, within the two age groups each participant read nine texts in a unique order. To furthermore avoid confounding effects from presenting the same three text items on a display, we used two different combinations of text items. For example, when a particular non-fiction text and another fictional text item were presented on the same medium in list one, they would not be presented on the same medium in the second list.

The presentation of a text on the digital reading devices started with a blank page containing a small black rectangle in the upper left corner, which participants were instructed to fixate. They were then informed to press a button on a gamepad to start reading. The blank page was then replaced with a text page. After having finished reading, participants pressed a button to proceed to the next blank page. Note that we used the button presses to send triggers on-line to the recording software of both the EEG and the eyetracking system for all reading devices.

Due to the inherently non-digital nature of the paper page, we made the following changes to the procedure described above. All sheets of paper were carefully filed on a music stand with the calibration page on top, and fixed with a rubber band at the bottom and a metallic clip at the top of the page. The texts were printed on high-quality paper (160 g/m^2^). For the calibration, all calibration points were printed on a paper page and indexed with subscript numbers. Similar to the two digital reading devices, the pacing interval during the calibration was 1000 ms. The participant was informed orally by one of the experimenters about the calibration point which he/she should be fixating. As with the digital reading devices, the calibration routine was repeated in the case of poor performance. Again, participants pressed a button to indicate that they were ready to start reading the next page. Here, the black rectangle appeared on the same sheet of paper as the text, but the text was hidden behind the previous page (either the calibration page or another text page). When the previous page was removed, the participant continued to fixate the black rectangle and pressed a button before beginning to read the text.

After the participant had read all three texts on a reading device, there was a short break during which the devices were exchanged and comprehension questions were presented (two per text; six in total per device). Comprehension questions probed contents from different pages of a text (literal, sentence-level messages in all cases) and required either a yes or a no answer. Comprehension probes were declarative sentences, which either described a part of the text correctly or, when a ‘no’-answer was required, replaced one word occurring in the text. For example, in one fiction text, the contents of the sentence ‘Ungern denkt Johnnie allerdings an Birte zurück, die vor Jahren vergaß, den Käfig der Lamas abzusperren.’ ('Johnny reluctantly remembered Birte who, years ago, forgot to close the lama cage.') was probed via ‘Birte vergaß vor Jahren, den Käfig der Löwen abzusperren.’ ('Years ago, Birte forgot to close the lion cage.'; requiring a ‘no’-answer) One experimenter read the questions out loud and noted the participant’s answer.

After the experiment, participants completed a short questionnaire, which contained questions about their usual reading behaviour and how they experienced the different reading devices. One experimental session – including instructions, EEG preparation and the post-experimental questionnaire – lasted approximately 2.5 to 3 hours.

### Data analysis

Power spectra were calculated per participant using a Fast Fourier Transform (FFT) on the EEG data from the first two pages read for each text. A Hanning-Window was used for FFT calculation and each page was treated as a single trial. The length of the EEG window used for the FFT analysis thus depended on participants' reading times per page, amounting to the following mean values (in seconds with standard deviations in parentheses; for number of EEG sample points multiply by 1000): young participants, tablet: 22.54 (5.29); young participants, e-reader: 23.00 (4.97); young participants, book page: 23.00 (4.71); older participants, tablet: 23.62 (6.56); older participants, e-reader: 27.76 (8.17); older participants, book page: 26.57 (7.69). We excluded the third page from all analyses as its text length varied considerably (see above). The analysis was conducted using the mean values for pages one and two.

We analysed activity (voltage density) in the theta frequency band, defined individually with reference to a participant's individual alpha frequency (IAF) as IAF-6 Hz to IAF-4 Hz [46] (for a discussion of the relevance of the IAF for language processing, see [47]). Thus, based on IAF (mean: 10.16; range: 8.44–12.18) and individual reading times, our analysis was based on an average of 100 (range: 58–174) oscillatory cycles per page for our lower cutoff frequency and 149 (range: 105–230) for our higher cutoff frequency. In order to avoid contamination of the EEG signal due to eye movements in the concurrent EEG-eyetracking setup used here, theta voltage density was calculated using the mean of the posterior electrodes (Pz, P3, P4, O1, O2) [31].

For the fixation time analysis, we first scanned the pages for blinks and for fixations that were not due to normal reading behaviour (e.g. fixations that resulted from participants anticipating the next black rectangle) using the EyeDoctor software (http://www.psych.umass.edu/eyelab/software/). Such events were excluded from further analysis (affecting<1% of all fixations in total). The remaining fixations (excluding blinks) were then summed to yield the final reading time per page.

All of our results were analysed statistically by means of linear mixed effects models [48] which were calculated using the lme4 package [49] in R. Prior to model fitting, outliers were removed by removing data points that were above or below the following thresholds: 1.5 inter-quartile ranges above the third or below the first quartile [50]. In all cases, model fitting proceeded as follows. We first fit a base model containing the fixed factors MEDIUM (reference level: tablet) and GROUP (reference level: young adults) plus additional predictors where appropriate and their interactions and random intercepts by participant. In a second step, we examined whether the additional inclusion of random slopes by participant and medium improved model fit as determined by likelihood ratio tests. Once the best-fitting random effects structure had been determined, we proceeded to determine the minimal adequate fixed effects structure by means of model simplification (i.e. successive removal of non-significant fixed effects from the model without a significant worsening of model fit [48], [50]). All figures were created using the ggplot2 package [51].

## Results

### Behavioural data: Comprehension questions

**Figure 1:**
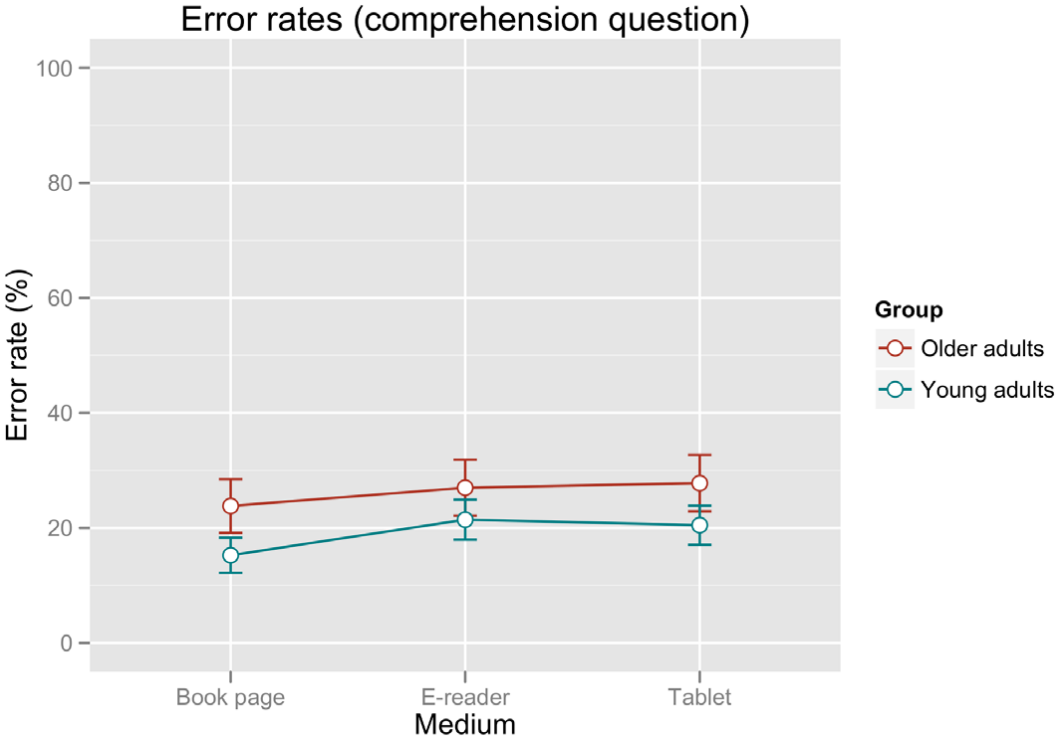
Mean error rates (%) for the comprehension questions in the present study. Error bars represent the standard error of the mean calculated for the within-participants factor MEDIUM according to the procedure outlined in [60].

Figure 1 shows the error rates for the comprehension questions per participant group and reading medium. Visual inspection of the figure suggests that the elderly participants showed a slightly higher error rate than the younger participants, but no differences between reading devices were apparent for either group.

This impression was confirmed by the statistical analysis, which was performed using logit mixed models for categorical data [52]. A model including random slopes per participant and medium showed no significant improvement over the base model including only random intercepts by participants (χ^2^(2) = 0.23, p>0.89). Successive simplification of fixed effects revealed that the minimal adequate model was one including (in addition to random intercepts by participant) only an intercept and the fixed factor GROUP and confirmed that error rates were higher for the older group (main effect of GROUP, estimate: 0.41, standard error: 0.17; p<0.02).

In summary, the analysis of the comprehension questions showed no effects of reading medium, but a slightly higher general error rate for elderly as opposed to young participants.

### Subjective judgements

**Figure 2:**
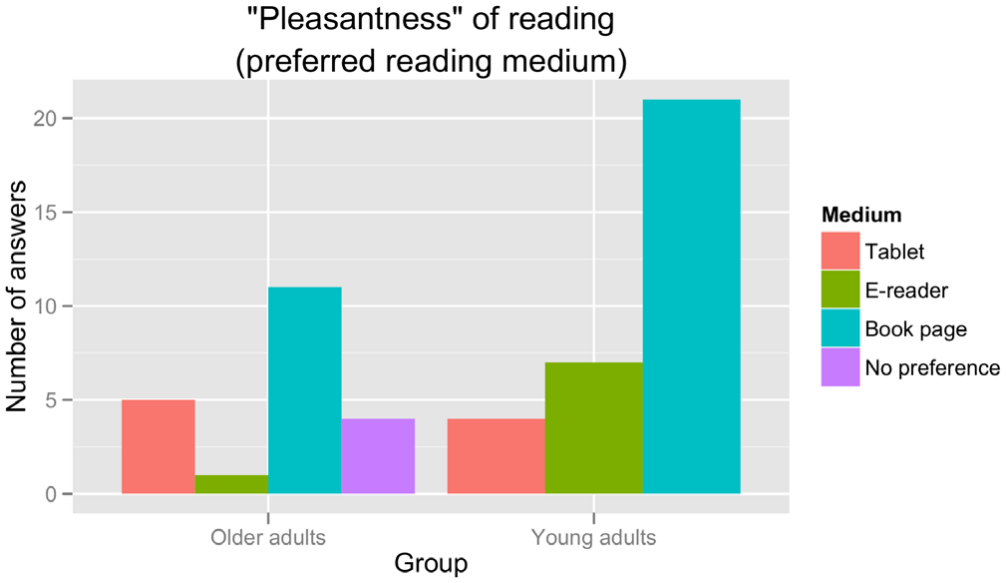
Ratings for the pleasantness of reading (choice of preferred reading medium) in absolute numbers of answers. Note that two participants were excluded from this analysis: one did not provide an answer, while the other chose two reading devices (tablet computer and book page).

**Figure 3:**
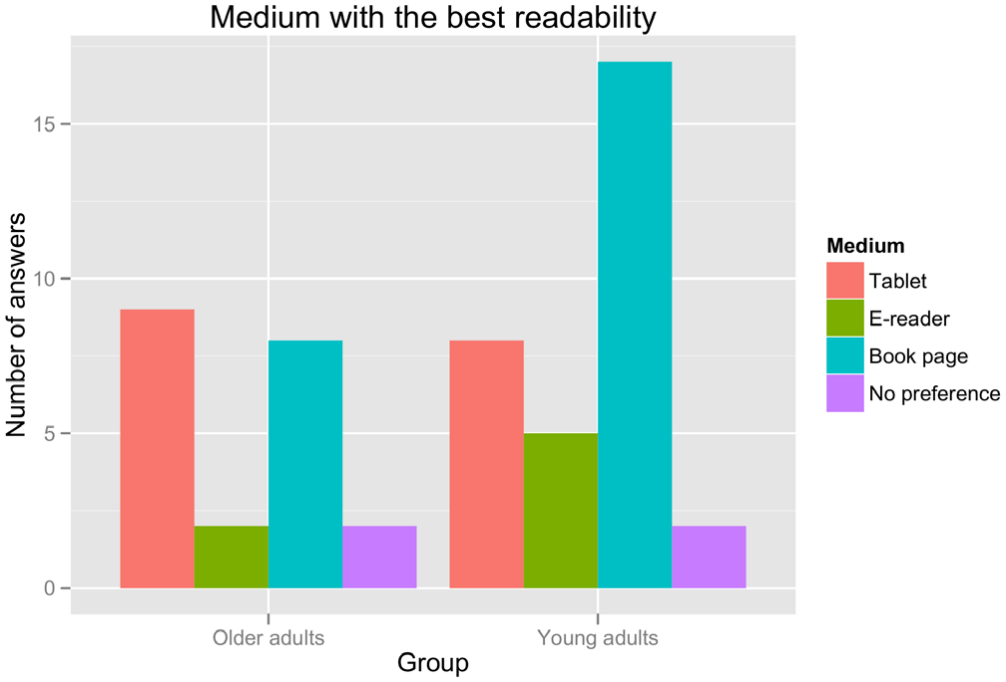
Ratings for the best readability / legibility across the different reading media in absolute numbers of answers. Note that three participants were excluded from this analysis: one did not provide an answer, while the other two chose two reading devices (tablet computer and book page).

In order to examine participants' subjective impressions regarding the different reading devices used in the present study, we analysed two questions from the debriefing questionnaire: (a) ‘pleasantness’ of the reading experience during the experiment, asking participants to choose their preferred reading device; and (b) ‘readability’ / legibility for the different devices, asking participants to choose the device with the highest perceived ease of reading / legibility of the text. The results for these two questions are shown in Figures 2 and 3, respectively.

As is apparent from Figure 2, both younger and older participants showed an overwhelming preference for the book page when asked to choose their preferred reading medium (book page versus tablet: χ^2^(1) = 13.71, p<0.001; book page versus e-reader: χ^2^(1) = 15.24, p<0.0001; no difference between tablet and e-reader: χ^2^(1) = 0.06, p>0.8). For readability ratings (Figure 3), by contrast, an advantage begins to emerge for the tablet computer as opposed to the e-reader (book page versus tablet: χ^2^(1) = 1.50, p>0.21; book page versus e-reader: χ^2^(1) = 10.13, p<0.01; tablet versus e-reader: χ^2^(1) = 4.17, p<0.05) and this pattern is driven particularly by the ratings of the older adults.

In summary, the results of our debriefing questionnaire replicate previous reports of subjective preferences for traditional in comparison to digital reading media (e.g. [1]). They also suggest, however, that there may be more fine-grained additional differences between different types of electronic reading devices.

### Online measures

In analysing online measures of reading effort, we proceeded as follows. We first computed separate analyses for fixation durations and theta activity, respectively, before going on to examine possible correlations between eye fixations and EEG measures.

#### Fixation durations

**Figure 4:**
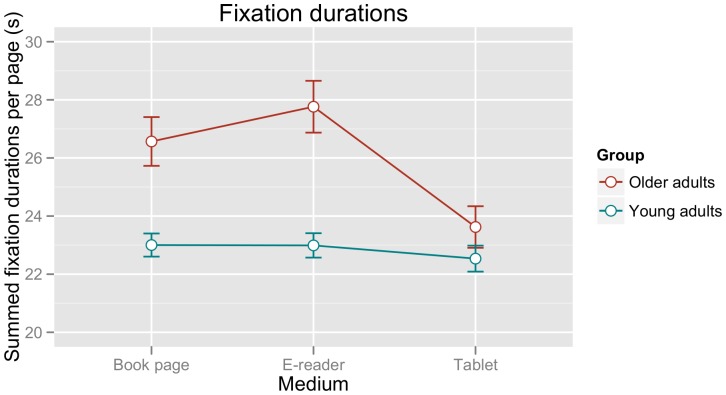
Mean summed fixation durations per page of text (in seconds). Error bars represent the standard error of the mean calculated for the within-participants factor MEDIUM according to the procedure outlined in [60].

Figure 4 shows mean summed fixation durations per page of text for each participant group and each of the three reading devices. The figure indicates that, while fixation durations did not differ across devices for the younger group, older participants showed slowed reading behaviour for the book page and e-reader in comparison to the tablet computer.

The statistical analysis indicated that a linear mixed effects model with random slopes per medium and participant (in addition to the fixed factors MEDIUM and GROUP) showed a significantly better fit to the data than a base model including only random intercepts by participant (χ^2^(5) = 95.77, p<0.0001). Thus, random slopes were included in all further model fits. Specifications for the minimal adequate model are shown in Table 1, which reveals significant interactions between GROUP and reading MEDIUM. In order to examine the source of these interactions in more detail, we computed separate mixed model analyses for each of the participant groups. These showed no effects of MEDIUM for the younger adults (all ts<1.20). For the older adults, by contrast, effects of MEDIUM were observed for both the e-reader (estimate: 4.14, std. error: 0.94, t-value: 4.39) and book page (estimate: 2.94, std. error: 0.51, t-value: 5.72).

**Table 1:** Parameters for the fixed effects of the minimal adequate model (see text for details) of the summed fixation durations per page in the present study.

Effect	Estimate	Std. error	t-value
Intercept	22.54	0.91	24.80
Group:Older adults	1.09	1.48	0.73
Medium:E-reader	0.45	0.54	0.84
Medium:Book page	0.47	0.44	1.05
Group:Older adults * Medium:E-reader	3.68	0.88	4.18
Group:Older adults * Medium:Book page	2.48	0.73	3.41

The analysis of the fixation durations thus shows that younger and older participants perform differently when using different reading devices. While the young adults showed comparable fixation durations for all three reading media, fixation durations for the older adults were longer for both the e-reader and the paper page in comparison to the tablet computer.

#### EEG activity (theta band)

**Figure 5:**
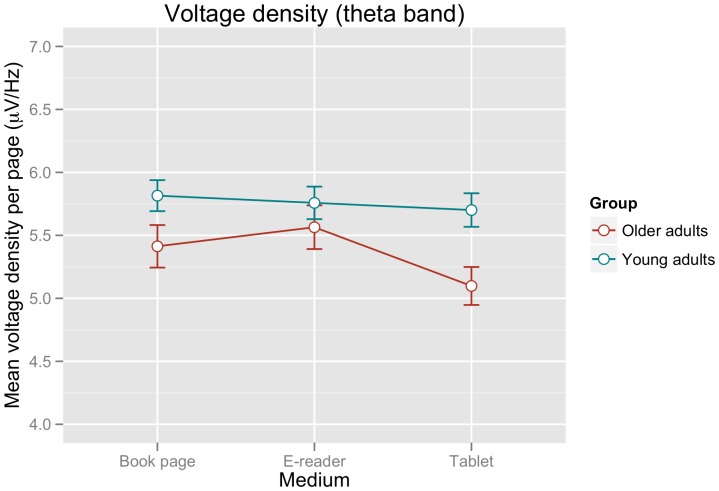
Mean voltage density measures for the EEG theta band (μV/Hz) per page of text. Error bars represent the standard error of the mean calculated for the within-participants factor MEDIUM according to the procedure outlined in [60].

A very similar pattern of results was apparent for EEG theta band activity, as shown in Figure 5 and Table 2. Table 2 again shows the minimal adequate linear mixed effects model including random slopes by participant and medium (justified by a significant improvement over a model involving only random intercepts by participant: χ^2^(5) = 14.67, p<0.02). Separate analyses per group revealed no effects of MEDIUM for the younger adults (all t-values<1.20), while the older adults showed effects of MEDIUM for both the e-reader (estimate: 0.47, std. error: 0.17, t-value: 2.70) and book page (estimate: 0.33, std. error: 0.10, t-value: 3.22) in comparison to the tablet computer.

**Table 2:** Parameters for the fixed effects of the minimal adequate model (see text for details) of the theta band voltage density per page in the present study.

Effect	Estimate	Std. error	t-value
Intercept	5.70	0.23	25.10
Group:Older adults	−0.60	0.37	−1.63
Medium:E-reader	0.06	0.11	0.50
Medium:Book page	0.12	0.09	1.23
Group:Older adults * Medium:E-reader	0.41	0.18	2.22
Group:Older adults * Medium:Book page	0.22	0.15	1.40

Similarly to the results for the fixation durations, the analysis of voltage density in the theta band showed comparable values for each reading device for the younger adults, but an increase in theta activity for the e-reader and book page in comparison to the tablet computer for the group of older adults.

#### Fixation durations as predictors for theta band activity

**Figure 6:**
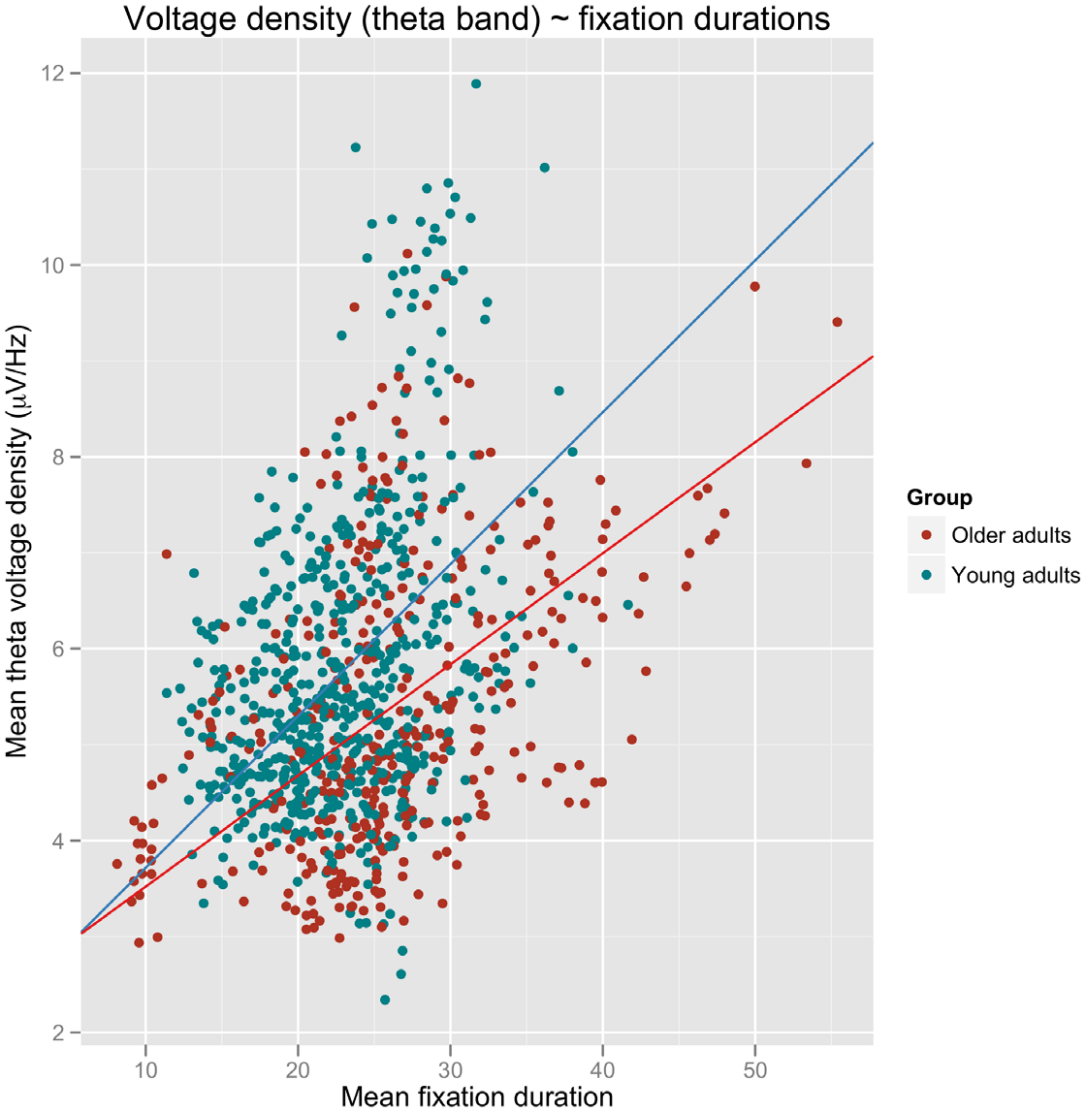
Mean voltage density measures for the EEG theta band (μV/Hz) per page of text as a function of mean summed fixation durations (s) per page. Regression lines represent the parameters of the minimal adequate mixed effects models for the two groups (see text for details)

In view of the similar data patterns for fixation durations and theta band activity and in accordance with our assumption that both measures should serve as good correlates of online information processing effort, we performed a final analysis in which we directly examined the relationship between the two. This is depicted graphically in Figure 6, while Table 3 shows the model specifications for the minimal adequate mixed effects model including FIXATION TIME as a continuous predictor (in addition to the fixed effects GROUP and MEDIUM) and theta activity as the dependent variable. The model once again includes random slopes by participant and medium as these led to a significant improvement in fit over a model including only random intercepts by participant (χ^2^(5) = 15.25, p<0.01). As is apparent from Table 3, fixation times and theta activity indeed show a correlation, which, however, also seems to depend on participant group as indicated by the GROUP x FIXATION TIME interaction. Separate analyses for each GROUP showed significant effects of FIXATION TIME for both younger (estimate: 0.16, std. error: 0.01, t-value: 14.03) and older adults (estimate: 0.12, std. error: 0.01, t-value: 11.99).

**Table 3:** Parameters for the fixed effects of the minimal adequate model (see text for details) of theta band voltage density, including fixation durations as a continuous predictor variable.

Effect	Estimate	Std. error	t-value
Intercept	2.12	0.32	6.56
Group:Older adults	0.26	0.48	0.53
Medium:E-reader	−0.01	0.08	−0.15
Medium:Book page	0.03	0.06	0.42
Fixation time	0.16	0.01	14.64
Group * Fixation time	−0.05	0.01	−3.16

In summary, the joint analysis of EEG and eye movement measures showed that fixation durations reliably predict modulations in theta activity: theta voltage density increases with increasing fixation times. This effect was observable across both participant groups, but slightly more pronounced for the young adults.

## Discussion

We have presented a concurrent EEG-eyetracking study that examined text reading on digital media (a tablet computer and an e-reader) in comparison to a paper page in both young and elderly adults. While the young adults showed no difference in fixation durations and EEG theta band activity across the three different reading devices, results for the older adults revealed faster fixation durations and reduced theta voltage density for reading with a tablet computer in comparison to both the e-reader and the paper page. These differences cannot be explained in terms of comprehension accuracy, which did not differ across reading media for either group. They also do not correspond to participants' subjective judgements regarding their reading experience, in which they showed an overwhelming preference for the paper page in terms of pleasantness ratings and (to a somewhat lesser degree) readability. In addition, they do not reflect the relative resolution differences between the three media, since none of our measures showed the paper page<tablet<e-reader gradation predicted by resolution differences. Finally, across all participants and media, our findings showed a correlation between fixation durations and theta activity, with longer fixation times correlating with higher theta voltage density.

Perhaps the most striking finding of the current study is the complete lack of a correspondence between the offline measures collected (comprehension accuracy and subjective ratings) and the online measures of reading effort. Thus, though participants stated that they preferred the book page over the electronic reading devices, none of the quantitative online measures collected support that reading was more effortful for the digital media. To the contrary, older participants in fact benefitted from the use of a tablet computer, as shown by both faster reading times and lower theta voltage density for this device as opposed to the other two. Crucially, this observation cannot be attributed to a speed-accuracy tradeoff, since comprehension rates did not differ across the three reading media. Our results thus indicate that negative subjective assessments of readability for e-books or other digital texts (e.g. [1]) are not a reflection of real-time information processing demands. While we can only speculate with regard to the underlying source of these – highly consistent – subjective impressions, it appears reasonable to assume that they may be tied to the important status of traditional (printed) books as part of our cultural heritage. Lack of familiarity with digital reading, by contrast, does not seem to suffice as an explanation: firstly, our behavioural results mirror those published five years ago by Rowlands et al. [1] in spite of the rapid increase in e-books during that time span; secondly, the fact that our younger participants preferred the traditional reading experience to a (numerically) higher degree than our older participants (see Figures 2 and 3) also speaks against a familiarity-based explanation. The present findings thereby suggest that the skepticism towards digital reading media that was described in the introduction may reflect a general cultural attitude towards reading in this manner rather than measurable cognitive effort during reading.

A second intriguing finding of the present study was the reduction of online reading effort for older adults when reading with a tablet computer in comparison to an e-reader or a book page. Why should this be the case? The most obvious factor that sets apart the tablet from the other two reading devices is the backlighting of the display, thus increasing the contrast between text and background. Indeed, previous eyetracking research has shown that changes in contrast modulate fixation durations. For example, Reingold and Rayner [53] found that a reduced contrast leads to longer fixation times for a critical word – amounting to an increase of first fixation times by 60 ms and of gaze duration (i.e. the sum of all fixations on the critical word before the first saccade to another word) by 120 ms. This result was replicated in a subsequent study [54], which further showed that a reduced contrast leads to a lower probability of word skipping. Thus, there is good evidence to support the relationship between contrast and reading times, thereby providing a potential explanation for differences between the tablet computer and the other two reading devices.

This does not yet explain, however, why these differences were only observed for the older readers. In this regard, we propose that older adults may be generally more susceptible to effects of text discriminability. Importantly, reduced contrast-sensitivity with increasing age is well documented (e.g. [55]; for discussion of age-related changes in sensory function in the context of a reading model, see also [56]). While we are not aware of any reading studies that have examined the relationship between contrast and age directly, there is evidence to suggest that older readers are affected more strongly by other types of perceptual influences during reading. Rayner et al. [57], for example, employed a ‘font difficulty’ manipulation by comparing Times New Roman and Old English fonts and observed a larger font effect for older readers (i.e. while both younger and older readers showed longer fixation durations for the more difficult font, this effect was more pronounced for the older readers). In sum, while this claim will clearly require further investigation in future research, the assumption that older readers benefit from the added contrast produced by backlit text parsimoniously accounts for the reduced fixation durations for the tablet computer in this age group. Furthermore, in view of the tight interrelationship between fixation times and theta band activity observed in the present study, this explanation potentially carries over to our EEG results as well.

This brings us to the third major finding of the current experiment, namely the correlation observed between fixation times and theta band voltage density. On the one hand, this correlation strengthens the conclusions drawn above with regard to (a) the dissociation between subjective impressions regarding preferred reading media and objective online reading effort, and (b) the benefit of reading on a tablet computer for older adults, since both online reading measures show a very similar pattern of results. On the other hand, the finding is also interesting in and of itself, i.e. independent of the discussion regarding different reading devices, since direct correlations between eye movement measures and event-related potentials have remained somewhat elusive in the past [8], [9]. Connections between eyetracking and frequency-based EEG measures, as observed here, have not been examined directly to date, though we have hypothesised in past research that they may provide a more promising means of correlating the two methods in reading [24]. The present findings provide initial converging support for this assumption. The nature of the correlation, however, clearly requires more detailed investigation in future research in order to determine, for example, whether it can also be observed at a more directly stimulus-locked (i.e. word-by-word) level and whether phase modulations in oscillatory EEG activity can be linked to fixation durations in eye movement measures (as proposed in [24]).

Importantly, the correlation between fixation times and theta activity was observed across both participant groups, thus showing that the absence of medium-related effects in the younger adults did not result from an overall null effect. Rather, both older and younger adults showed variable fixation durations and concomitant changes in theta activity during reading in the current study, but these changes were not conditioned by reading medium in the younger group.

To conclude, the present findings provide no evidence to support the assumption that online reading effort increases when people read on digital devices as opposed to paper. To the contrary, they suggest that digital media may even provide advantageous reading conditions under certain circumstances, notably when they provide improved discriminability for older readers. Of course, this is a only a first result that will require corroboration and further investigation in future research (e.g. by testing whether it extends to more prolonged periods of reading on a particular device). Nevertheless, our data show a robust dissociation between two separate online measures of reading effort - fixation durations and EEG theta band activity - on the one hand and subjective impressions of pleasantness of reading and readability on the other. This suggests that the overwhelming public opinion that digital reading media, though convenient, reduce the pleasure of reading is a cultural rather than a cognitive phenomenon. From this perspective, the subjective ratings of our participants (and those in previous studies) may be viewed as attitudes within a period of cultural change. To some degree, they could thus be likened to the phenomenon of language change, which is viewed as a natural reflection of language as a dynamical system from a scientific perspective, but is often popularly characterised as a degradation of language (for examples with regard to German, see [58], [59]). Our findings thus provide yet another piece of evidence that our cognitive and neural processing of the changing environments around us must be dissociated from our evaluation of these changing circumstances.

## Supporting Information

Text S1 **Summary of relevant facts regarding the sale of e-books and recent studies regarding German readers' acceptance of digital reading devices.**
(DOC)
