# Supplementary material for: Subjective Impressions Do Not Mirror Online Reading Effort: Concurrent EEG-Eyetracking Evidence from the Reading of Books and Digital Media
Source: PLoS One. 2013 Feb 6;8(2):e56178. doi: 10.1371/journal.pone.0056178 (PMC3566074; doi:10.1371/journal.pone.0056178)
Supplement: Text S1 — Summary of relevant facts regarding the sale of e-books and recent studies regarding German readers' acceptance of digital reading devices. (DOC) [file pone.0056178.s001.doc]

Text S1. Summary of relevant facts regarding the sale of e-books and recent studies regarding German readers' acceptance of digital reading devices.

In April 2011, Amazon.com announced that sales of e-books outnumbered those of print books (with 105 books for the Kindle e-reader being sold for every 100 hardcover or paperback books; <http://www.nytimes.com/2011/05/20/technology/20amazon.html>). Similar figures have recently been released for the United Kingdom: in August 2012, Amazon.co.uk sold 114 digital books for every 100 print books (<http://phx.corporate-ir.net/phoenix.zhtml?c=251199&p=irol-newsArticle&ID=1722449>).

Nevertheless, in a 2012 study of German readers (n=7202; [http://www.boersenverein.de/sixcms/media.php/976/E-Book-Studie%202012%20PRESSEMAPPE_print.pdf](http://www.boersenverein.de/sixcms/media.php/976/E-Book-Studie 2012 PRESSEMAPPE_print.pdf)), 72% of people questioned agreed with the statement "I do not want to read from a display / monitor" (with 41% stating that they "agree completely" with this statement and 31% stating that they "agree to some degree"). Moreover, 82% of people questioned agreed with the statement "I love printed books too much; an electronic device does not produce the same kind of reading result" (55% agreed completely; 27% agreed to some degree).
